# In medical research, what appears to be intuitive and sensible might be erroneous: percent change from baseline

**DOI:** 10.3325/cmj.2020.61.569

**Published:** 2020-12

**Authors:** Vladimir Trkulja, Pero Hrabač

**Affiliations:** 1 Department of Pharmacology, Zagreb University School of Medicine, Zagreb, Croatia *vtrkulja@mef.hr*; 2 Department of Medical Statistics, Epidemiology, and Medical Informatics, Andrija Štampar School of Public Health, University of Zagreb School of Medicine, Zagreb, Croatia

In the previous issue of the *Croatian Medical Journal* (1) we stressed the importance of methodological knowledge for conducting and understanding sensible medical research and to point out that medical expertise (another pivotal prerequisite) and common sense/intuition cannot compensate for a lack of methodological knowledge. We outlined an imaginary research study to illustrate several (out of an enormous number of possible) situations in which apparently sensible choices turned out to be flawed, resulting in misleading results and interpretation. In the present issue, we provide another simplified outline of the main points of an imaginary research study (but composed of elements of true research, published or not, that we have come across over the years) to depict another set of apparently sensible but erroneous methodological procedures. We stay within the setting of randomized controlled trial (clinical experiment – units of observation are human beings) as we presume that the depicted concepts might be more “catchy” to medical doctors when presented within a framework dealing with human subjects. The same concepts, however, are fully applicable to any kind of an experiment: units of observation might be molecules in a test tube, cells in a cell culture or laboratory animals. In addition, although methodologically rather complex, experiments are still less demanding than observational studies intended to detect independent associations (yet alone causal relationships). We use the term “treatment” to depict a presumed cause (to a consequential outcome), which in clinical trials is a therapeutic or a prophylactic intervention (but could be a range of different things in different types of experiments), and we use the term “placebo” to depict a control that conveys the same “idea of treatment” but is biologically inert on its own – a setting that (if things are done adequately) has a high potential to accurately identify and quantify a treatment effect.

Oxidative stress has been implied to play a role in a number of disorders and is considered particularly important in periodontitis (chronic inflammation of the gum, eventually leading to bone resorption and loss of teeth), although the condition is multifactorial (2). Cigarette smoking, diabetes, obesity, chronic inflammatory diseases (eg, rheumatoid arthritis), and several other known risk factors for periodontitis in part exert their effect by increasing local oxidative stress (2). On the other hand, several natural products typically used as food supplements with antioxidant activity show beneficial effects in certain animal models of the disease (2). A research group conceived a mix of such compounds in the form of a gel that can be delivered locally to the periodontium. As an initial step in its clinical evaluation, they wish to see whether it could reduce the level of oxidative stress in the affected tissue [see (3) for a method to quantify total reductive potential of tissue samples] – a somewhat different method from a rather common approach of measuring antioxidant capacity/oxidant status in the saliva of such patients (4). To achieve this, they plan a randomized placebo-controlled trial (placebo = gel without presumed active substances) in newly diagnosed patients with periodontitis. No previous studies in this setting used this quantification method, and generally there is little input on what level of variability or what effect size one should expect when it comes to the quantification of total reductive tissue capacity. Under such circumstances, it is difficult to calculate the sample size for the trial, but it appeared sensible that enrolling around 10 participants to the treatment arm and 10 in the placebo arm should provide a reasonable insight into a possible treatment effect. To reduce heterogeneity, it is planned to include only men (avoid potential hormonal influence), moderate smokers (10 to 20 cigarettes a day), non-diabetic and non-obese participants, and generally participants free of metabolic or other serious systemic diseases. The planned procedure with consenting participants is as follows: at the start of the trial, participants (in groups of 10) are to be admitted to the research site between 07:00-07:30 am. A proper method of oral hygiene is to be demonstrated. Each participant will be provided a needed kit and then perform the procedure. Between 08:30-09:00, each participant will undergo another periodontal check, and three miniature tissue samples will be taken from the inflamed regions and frozen. Subsequently, participants will be randomized. All participants will be provided a kit for the practiced oral hygiene method. The treated group will receive a pack of the test gel to be administered twice daily by rubbing it on the gum, after having completed oral hygiene. The control group will receive a pack of a matching placebo gel with the same instructions. After 7 days of treatment, the procedure (oral hygiene, periodontal check, and tissue sampling from the same regions) will be repeated. The tissues will be analyzed by an analyst blinded to patients and treatment. The method yields arbitrary units of total tissue reductive capacity normalized to protein content (eg, per μg of proteins) – higher values mean higher reductive capacity.

Table 1 depicts fragments of (imaginary) reported data pertaining to baseline characteristics and to the outcomes of interest: a) baseline data refer only to the pre-treatment tissue total reductive capacity (TRC) and age; b) end-of-study data refer to the TRC values measured at the end of treatment and the outcome of interest is the “change in TRC from baseline.” Since TRC is reported in arbitrary units, the observed differences between treatment and placebo are not very intuitive for interpretation. Consequently, the authors also report “percent change in TRC from baseline”. Data in Table 1 require three comments.

**Table 1:** Fragments of (simulated) data from the imaginary trial pertaining to baseline – only age and total tissue reductive capacity (TRC) – and end-of-study outcomes: measured TRC, change in TRC vs baseline, and change in TRC expressed as “percentage change.” Data are mean ± standard deviation (min-max) and differences are mean differences with 95% confidence intervals, respective t-scores, and P-index*

	Treatment	Placebo	Treatment-Placebo
N	10	10	—
At baseline			
age (years)	51.5 ± 11.7 (40-80)	62.0 ± 14.0 (39-80)	-10.6 (-22.7, 1.6); -1.83; 0.083
TRC (arbitrary)	1.246 ± 0.302 (0.942-1.974)	1.098 ± 0.244 (0.635-1.420)	0.148 (-0.109, 0.406; 1.21;0.242
At trial end			
TRC (arbitrary)	2.013 ± 0.669 (0.927-2.919)	1.271 ± 0.278 (0.989-1.740)	0.742 (0.243, 1.241); 3.24; 0.007
change in TRC	0.767 ± 0.501 (-0.103, 1.436)	0.173 ± 0.190 (-0.151, 0.409)	0.594 (0.238, 0.949); 3.51; 0.002
TRC change as %	61.36 ± 39.96 (-10.02, 97.98)	18.03 ± 21.2 (-12.2, 55.73)	43.33 (13.30, 73.35); 3.03; 0.007

*Age was sampled from a normal distribution with mean 52 and standard deviation 11 for treated and with mean 61.5 and standard deviation of 13.5 for placebo patients. Values were randomly assigned to individual subjects. Baseline TRC values for treated and placebo patients were sampled from the same lognormal distribution. End-of-trial values were sampled from distributions with slightly shifted μ and σ, somewhat more shifted for treated than for placebo patients. Values were assigned in order as sampled.

1. *Statistical tests for baseline subject characteristics (covariates)*. The CONSORT statement (http://www.consort-statement.org/checklists/view/32-consort-2010/510-baseline-data) explicitly warns that performing statistical tests on baseline covariates to compare participants allocated to different treatment arms in RCTs is a poor practice that should be avoided. It is (quote) “*not necessarily wrong*”, but it is “*illogical…superfluous and can mislead investigators and their readers*.” Randomization [a set of different specific procedures or techniques see, eg (5),] is a procedure aimed to assign participants to treatments without a prejudice – that is, irrespective of their characteristics (covariates) at the start of an experiment. Randomization techniques are many, of different complexity, but the main purpose is always the same. Some, like the one applied in the outlined imaginary trial, enable a balance in the number of participants across treatment arms even if the total sample is small. But, more importantly, if done correctly [meaning: randomization plan/list was generated by a true randomization technique; investigators recruiting participants do not know and cannot guess the treatment to be assigned to the next patient], randomization enables exchangeability of participants in the trial. This means that participants assigned to one and the other arm/treatment (say, A and B) differ (on average) only in respect to the arm to which they were assigned – and this refers to their measured baseline covariates but also to all unmeasured covariates. It also means (but this extends far beyond the focus of the present column) that whatever difference in the outcome between A and B is observed, it would have been exactly the same had the participants randomized to A been randomized to B and *vice-versa*. A statistical test, for example, a *t* test used to compare treated and placebo patients for age (or TRC at baseline) in Table 1, is a formal test of an *a priori* hypothesis (the null hypothesis reading: treatment – placebo difference = 0). The resulting P-index depicts a probability (eg, *P* = 0.083, ie, 8.3%) of observing a difference equal to or larger than the one observed (in a large number of repeated tests in a large number of independent random samples of the same size drawn from the same population) under the condition that the null hypothesis is true – ie, under the condition that the only possible source of a difference is chance alone. But – we already know: this is an RCT, patients were randomized, so the only possible source of a difference is chance (assuming that randomization was adequately performed). Such a statistical test has no purpose at all and is, in logical terms, completely misplaced (6). The “urge” to perform tests of baseline covariates likely stems from a slight misunderstanding of the randomization process: even if performed perfectly, it does not necessarily need to result in a perfect balance of all measured covariates – by chance, some imbalance can always occur. This does not mean that randomization was not “successful” – the key is (i) appropriate method to generate the randomization schedule and (ii) appropriate method of the allocation schedule concealment from the recruiting investigators. In large trials, the balance is typically achieved, but not without exceptions. In small trials, particularly, imbalances can occur. Some other points contribute to the view about a lack of logic in using statistical tests for baseline covariates. In the outlined imaginary trial, no sample size was calculated (to achieve a desired power to detect an effect of a particular size), but sample size in RCTs is determined in respect to the outcomes of interest – not in respect to (possible) baseline imbalances between randomized arms! So, a “high P-index” for a test of baseline covariate could be simply due to the lack of power! For example, had this trial included 20 and 20 participants per arm, both baseline TRC and age differences at baseline, of the same size as observed, would have been associated with a P-index lower than the commonly used “threshold” alpha level of 5% (ie, *P* < 0.05). What would this change? In reverse, in very large trials even miniature differences between randomized arms, eg, like 0.6 years difference in age – can be associated with a *P* < 0.001. Does this change the conclusion that the age is well balanced? Finally, when many serial tests are performed (each at the type 1 error rate of 5%), this increases the probability that at least one null-hypothesis will be rejected by chance. Considering all of this – it is clear that conducting serial statistical tests for baseline covariates in an RCT makes no sense. But, how can it be misleading? The background is the same as behind the “urge” to do it: (untrained) common sense/intuition as opposed to methodological knowledge. One sees a test for a baseline covariate associated with a P-index >0.05 and concludes that this specific covariate should not be taken into account when estimating the treatment effect (because it was “not statistically significantly different between the arms at baseline”). For example, based on data in Table 1 (P-index for “age” at baseline = 0.083), the authors concluded that age can be disregarded in calculation of the difference between treatment and placebo regarding the end-of-study TRC. But – should age indeed be considered a factor not likely to influence the outcome (TRC level)? If we look at Table 1 more carefully we see that randomization was performed adequately, but, by chance, there was an average difference of almost 11 years between the two arms. Should this be disregarded? The fact is, actually, as follows: if there is a variable (eg, age, smoking status, some comorbidity, or any other) for which it is known or it is plausible to assume that it can substantially affect the outcome, then one should plan a stratified randomization: eg, age could be split into bands (eg, 20-45, 46-65, and >65), and a separate randomization list is generated for each age stratum – this way, a balance will be achieved. (This is one and very common possible way to deal with such a situation). In the current trial, this was not done, and, by chance, some imbalance occurred. Next, each such variable, stratified at baseline or not, balanced or not – should be accounted for in data analysis. In the particular case of the outlined imaginary trial this simply means that treatment-placebo differences should be calculated using a model that adjusts for age, and the difference will be a difference between adjusted (for age) mean TRC values at the end of the trial.

2. *Adjusting for the baseline value of a measure that is also an outcome of interest at the end of the trial*. This point in a way naturally connects to the previous one: in Table 1, the authors provided (i) end-of-treatment TRC values and compared them between treatments; (ii) differences in the end-of-treatment TRC vs baseline TRC in each arm – and compared them. These two outcomes report about the same thing: the treatment effect on the tissue TRC – is it not somewhat strange that, depending on how it is calculated, the t-score is a bit different (the amount of difference and the associated P-index)? Which of the two actually illustrates the treatment effect? TRC at baseline was generally similar between the two arms, and following common sense/intuition – the authors concluded that it should not be taken into account in treatment effect estimation. Erroneous! A factor that most strongly affects a certain value taken at a certain moment (eg, end of trial) is a value of this specific variable at some previous time – both end-of-study TRC (or any other outcome, eg, LDL level, blood pressure, blood glucose, depression severity on the HAM-A scale, etc) and differences at the end vs baseline are always strongly influenced by the baseline value of the variable – end-of-study scores or changes vs baseline should always be compared with adjustment for the baseline values, regardless of a potential “perfect balance at baseline”! Table 2 summarizes (i) end of study TRC scores and (ii) change vs baseline TRC scores – adjusted for age and baseline TRC values. Note: although adjusted mean values for the two outcomes (end-of trial TRC and difference in end-of study TRC vs baseline) differ, treatment difference (treatment-placebo) is always the same and so is the t-score and P-index. Note, also, that with adjustment for age and baseline TRC, the treatment effect is quite smaller than the one reported by the authors in Table 1.

**Table 2:** Data are adjusted (for age and baseline tissue reductive capacity [TRC]) means (standard error) per treatment arm and differences Treatment-Placebo in adjusted means with 95% confidence intervals, respective t-scores, and P-index

	Treatment	Placebo	Treatment-Placebo
TRC at end of trial (arbitrary)	1.899 (0.118)	1.385 (0.118)	0.514 (0.139, 0.889); 2.91; 0.010
Change in TRC at the end vs baseline	0.727 (0.118)	0.213 (0.118)	0.514 (0.139, 0.889); 2.91; 0.010

3. *Percent change vs baseline*. The last row in Table 1 presents “change from baseline expressed as percentage” – provided are summarized data per treatment arm and their difference. Note: (i) although reporting on the same thing as the outcome in the row above (change in TRC) the t-score and P-index are different; (ii) this way of looking at data may generate (a false) impression that the treatment effect is greater than it is: 61% increase in TRC in treated patients vs 18% increase in TRC (on average) in placebo arm – sounds like 3 times greater increase. This appears as something that a (superficial) “common sense” could suggest. But, this would be greatly erroneous and a huge overestimate of the treatment effect. The source of confusion lies with the fact that the authors followed “common sense/intuition” in their attempt to express “percent change” – each individual difference vs baseline was divided by the baseline and expressed as a “percent of the baseline value,” then these were averaged and compared. The catch is in the fact that ratios (ratios of proportions or odds, as well as ratios of values of continuous variables) are not symmetrical on a natural scale! Let us assume that one participant experienced a 2-fold increase in TRC – his ratio (of end vs baseline) would be 2.0. Let us assume that another participant experienced a 2-fold decrease in TRC – his ratio would be 0.5. The average of two ratios would be 2.5/2 = 1.25, suggesting an “average increase of 25%,” where in fact – there is no average increase: there is a case of a 2-fold increase and a case of 2-fold decrease, and the “average change” is zero! However, logarithmic transformation of the data returns symmetry (7). In the case of continuous variables, like TRC, this means that the measured values are first logarithmically transformed; then all the calculations are done using logarithms; a treatment-placebo difference in mean logarithmically transformed values is calculated; finally, this difference is exponentiated to obtain a ratio of geometric means – ratio of geometric means is commonly used in clinical trials in which change vs baseline is used as an outcome (8). Table 3 summarizes differences (adjusted for age and baseline TRC) at the end-of-trial vs baseline for treatment and placebo expressed as geometric means ratios (GMRs). It also provides an estimate of the difference of the two GMRs (ie, ratio of ratios): (i) on average, in the placebo arm, TRC at the end of trial was by 16% higher than at baseline (GMR = 1.16); (ii) on average, in the treatment arm, TRC at the end of trial was by 56% higher than at baseline (GMR = 1.56); (iii) relative increase in TRC with treatment was on average by 34% higher with treatment than with placebo (difference in two GMRs, ie, ratio of GMRs = 1.34; 1.06-1.68).

**Table 3:** Age and baseline tissue reductive capacity (TRC) adjusted geometric mean ratios (GMR) and 95% confidence intervals for end-of-study vs baseline in each treatment arm and a difference between the two GMRs (ie, ratio of the two ratios)*

	Treatment	Placebo	Treatment-Placebo
GMR end-of study vs baseline	1.56 (1.32-1.83)	1.16 (0.99-1.37)	1.34 (1.06-1.68); 2.67; 0.016

*A generalized linear mixed model (distribution = normal, link = identity, REML estimation) was fitted to ln-transformed TRC values with fixed effects: treatment, time (baseline or end-of-study), treatment*time interaction, age and baseline TRC (proc glimmix in SAS 9.4). GMRs are derived from the interaction term.

In the present trial, procedures that resulted from “following (untrained) common sense/intuition” (instead applying methodological knowledge) did not result in any substantially false conclusions – it is only that adequately estimated treatment effect is smaller than the one reported, but generally supports the view that the tested treatment indeed might reduce local oxidative stress. This could set a path for further testing of its potential usefulness in treatment of periodontitis. Unfortunately, however, even in published papers, (unrecognized) methodological flaws are rather common and result in consequentially erroneous estimates inducing unsubstantiated enthusiasm about “dramatically announced” new treatments, diagnostic tests, prognostic factors, disease causes, or modifiers. Medical expertise and common sense may not be sufficient in order to recognize such misleading reports.
